# Supplementary material for: Transcriptional Analysis of the Endostyle Reveals Pharyngeal Organ Functions in Ascidian
Source: Biology (Basel). 2023 Feb 3;12(2):245. doi: 10.3390/biology12020245 (PMC9953650; doi:10.3390/biology12020245)
Supplement: Supplementary file 1 [file biology-12-00245-s001.zip › SupplementalFigures.pdf]

Supplementary Figures

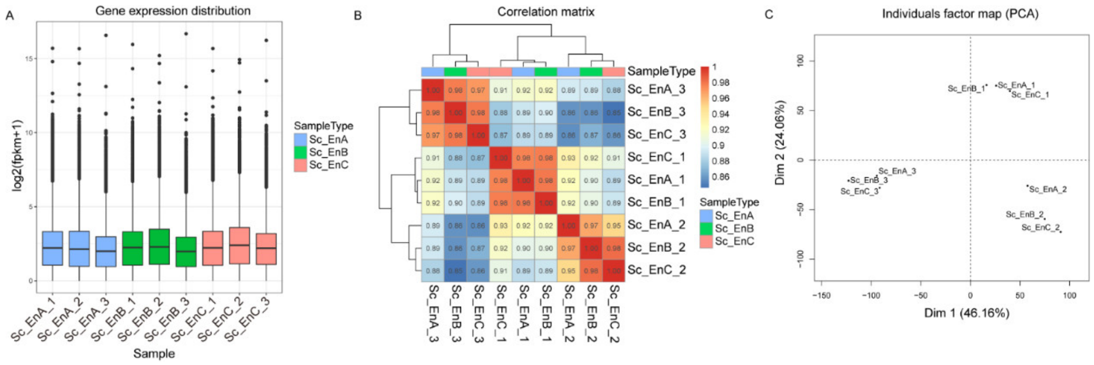

Supplementary Figure S1 RNA-seq data quality visualization. (A), Gene expression distribution of nine samples, eg. Sc_EnB_3 represents the second longitudinal segment on the third biological replicon. (B), Correlation matrix of nice samples. (C), Principal component analysis of nine samples.

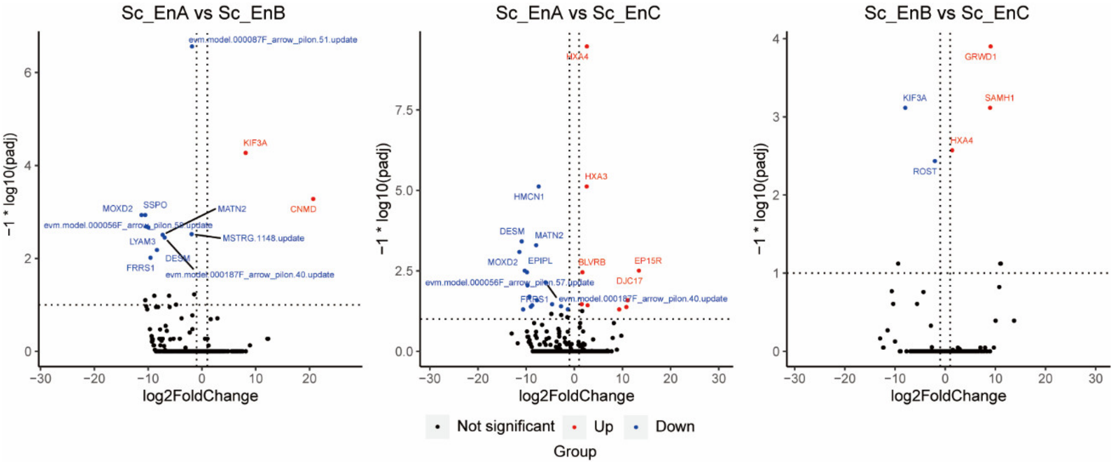

Supplementary Figure S2 Volcano plot of differential expressed genes among three segments of the endostyle. Red dots, blue dots, and black dots indicate up-regulated genes, down-regulated genes, and genes without significant differences respectively. Filtering threshold: log2FoldChange > 1, p adjust value < 0.05.

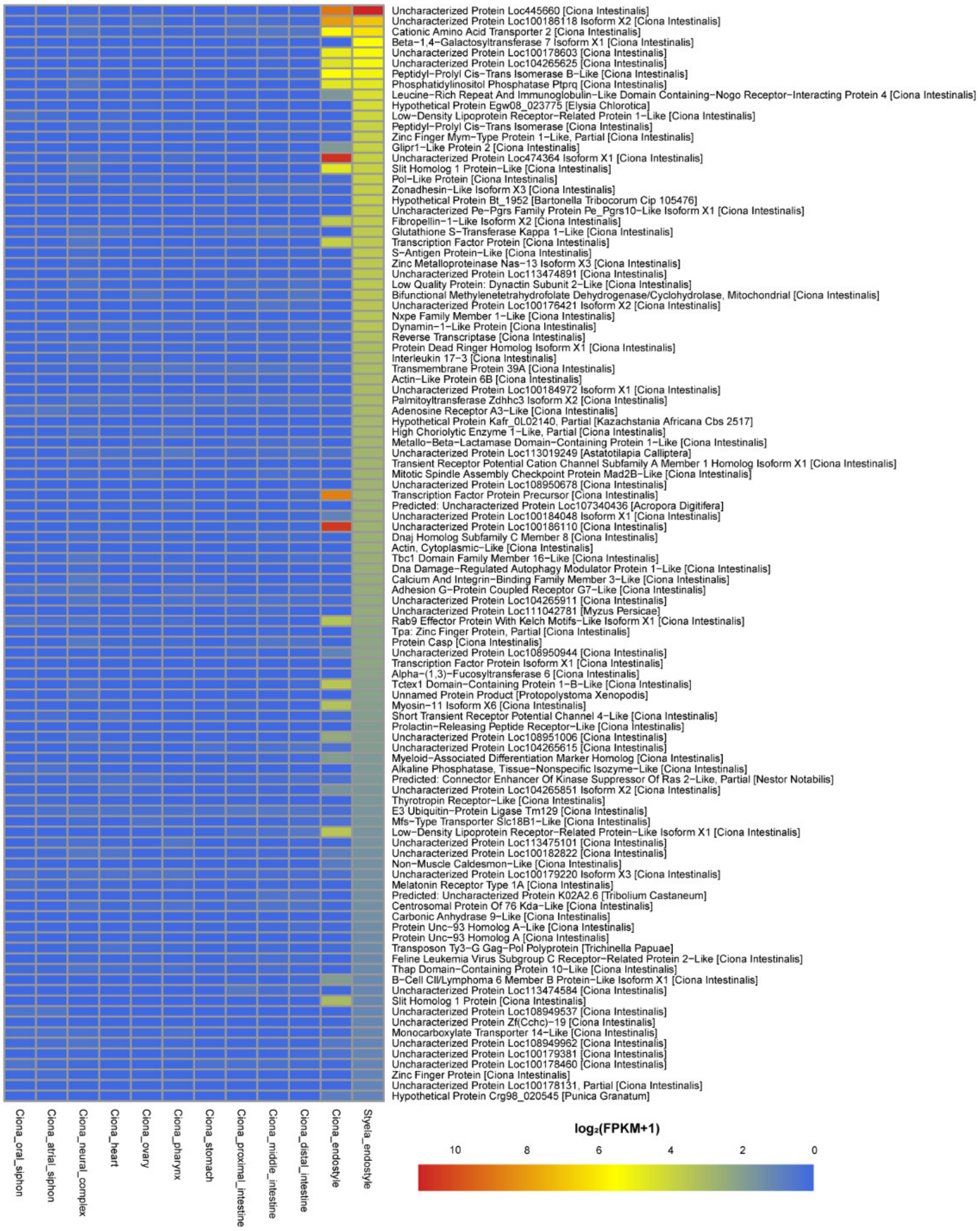

Supplementary Figure S3 Transcriptional expression profile of endostyle organ-specific gene (OSG) in ascidian organs. Homolog gene pairs of *Ciona* and *Styela* with expression value less than 0.5 in *Ciona* organs while expression values larger than 1 in *Styela* endostyle were defined as OSGs in the endostyle of *S. clava*.

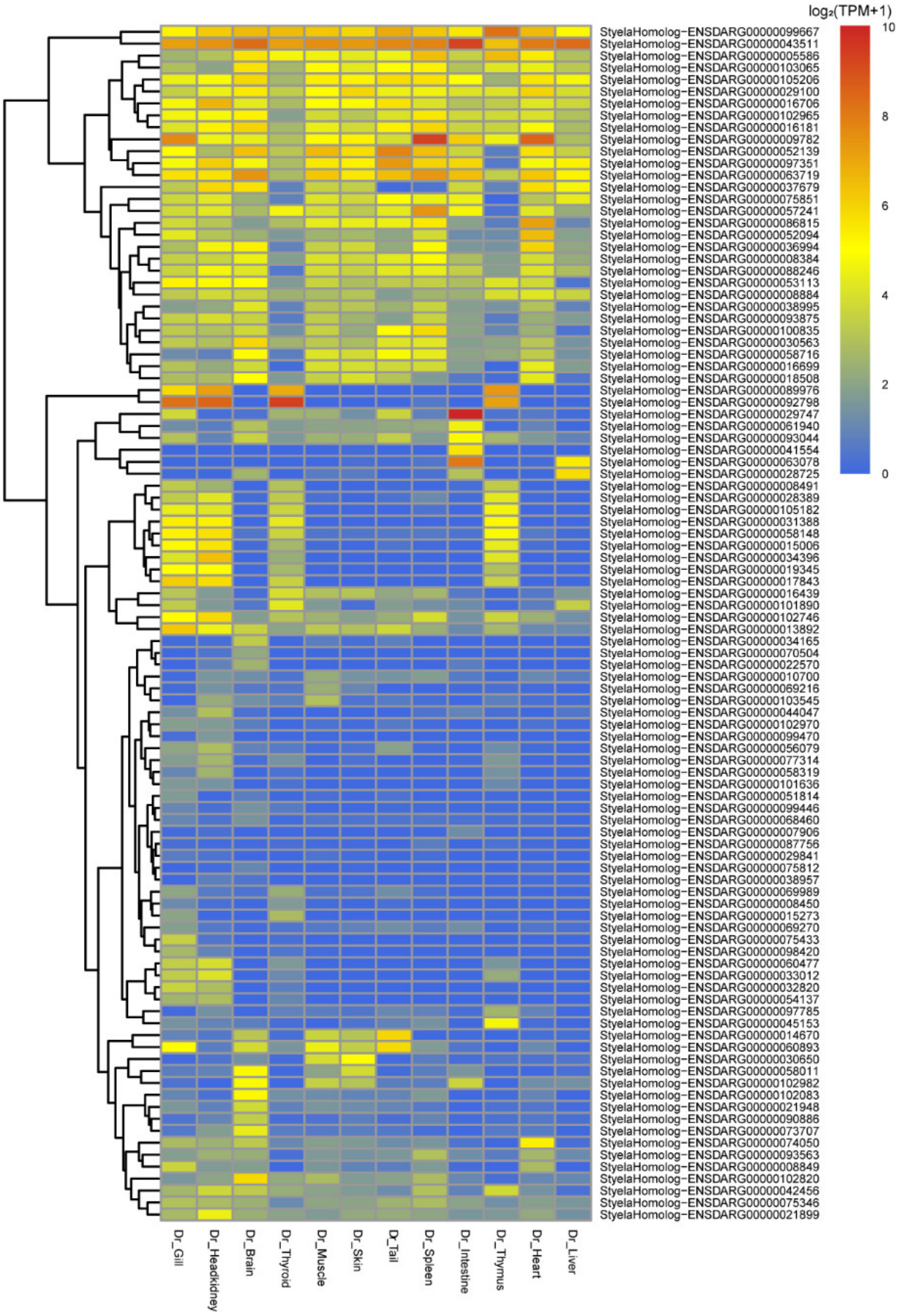

Supplementary Figure S4 Expression level of endostyle OSGs homolog in zebrafish organs. OSGs of endostyle of *Styela* were aligned against zebrafish reference contig (http://phylofish.sigenae.org/ngspipelines/data/gWFwrbaxnq/analysis/3q6S38NjnU/contigs.fasta.gz) and 95 of which hits homolog genes. The expression level of 95 homolog genes was plotted with pheatmap.

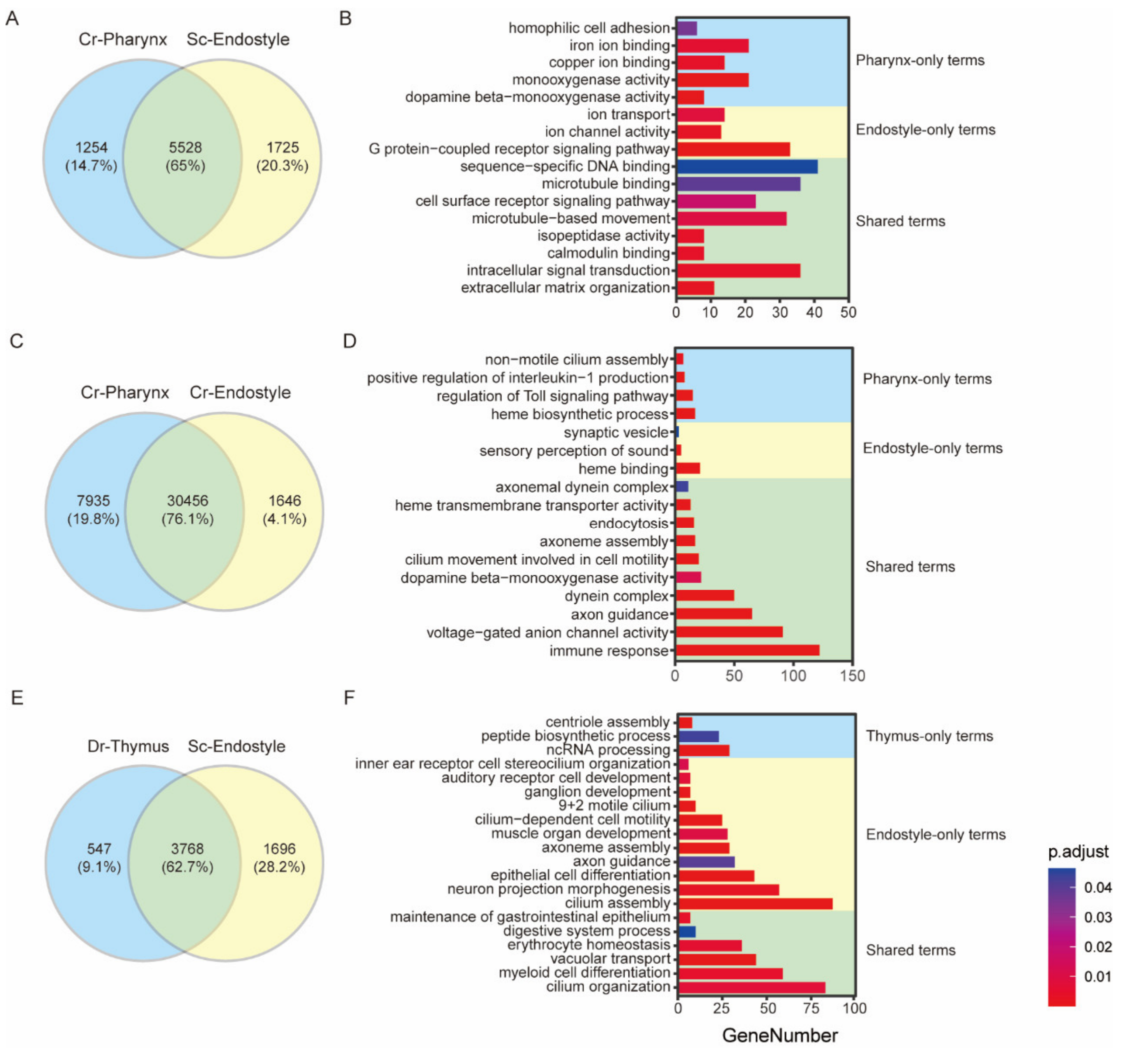

Supplementary Figure S5 Expression profile comparison between the endostyle and the pharynx/thymus. (A, C, E) Venn diagram is patronizing the shared and unique expressed genes in comparison parts, Cr-Pharynx and Sc-Endostyle, Cr-Pharynx and Cr-Endostyle, Dr-Thymus and Sc-Endostyle. (B, D, F) Gene ontology enrichment analysis for shared or organ-specifically expressed gene lists.
